# Cost Analysis of Prostate Cancer Care Using a Biomarker-enhanced Diagnostic Strategy with Stockholm3

**DOI:** 10.1016/j.euros.2024.05.010

**Published:** 2024-06-25

**Authors:** Olga Dianna McLeod, Thorgerdur Palsdottir, Jochen Walz, Derya Tilki, Alberto Briganti, Armando Stabile, Maria Nyre Vigmostad, Ashkan Mortezavi, Anas Elyan, Tim Dudderidge, Tim Govers, Henrik Grönberg, Hari Vigneswaran

**Affiliations:** a Department of Medical Epidemiology and Biostatistics, Karolinska Institutet, Stockholm, Sweden; b Department of Urology, Institut Paoli-Calmettes Cancer Center, Marseille, France; c Martini-Klinik Prostate Cancer Center, University Hospital Hamburg Eppendorf, Hamburg, Germany; d Division of Oncology, Unit of Urology, IRCCS Ospedale San Raffaele, Vita-Salute San Raffaele University, Milan, Italy; e Department of Oncology, Stavanger University Hospital, Stavanger, Norway; f Department of Urology, University Hospital Basel, Basel, Switzerland; g Department of Urology, University Hospital Southampton, University Hospital Southampton NHS Foundation Trust, Southampton, UK; h Medip Analytics BV, Nijmegen, The Netherlands

**Keywords:** Prostate cancer risk, Prostate-specific antigen, Stockholm3, Prostate cancer diagnostics, Risk prediction

## Abstract

**Background and objective:**

Building on previous research demonstrating better prostate cancer (PC) diagnostics via a biomarker-enhanced approach, this study focuses on cost analysis of PC care using the Stockholm3 test. We assessed the economic impact in European health care systems using real-world evidence for diagnostic outcomes and relevant costs.

**Methods:**

We evaluated two PC diagnostic strategies: (1) the conventional prostate-specific antigen (PSA) strategy with magnetic resonance imaging (MRI) and (2) PSA testing with a reflex to biomarkers at PSA ≥1.5 ng/ml in guiding decisions to perform MRI. Data from the Swedish National Prostate Cancer Register and Capio St. Göran Hospital provided real-world evidence, supplemented by health economic modeling. A comprehensive cost analysis was conducted using a Markov model for treatment pathways for four PC disease states and overall spending, for which costs from various European health care systems were used. A deterministic sensitivity analysis was performed across different cost and diagnostic scenarios.

**Key finding and limitations:**

The average cost for the four disease states was €2 182 for benign disease, €10 023 for low-grade disease, €13 073 for intermediate- to high-grade localized or locally advance disease, and €271 210 for metastatic disease. The overall spending was €358 239 (7.7%) lower per 1000 men tested in the biomarker-enhanced strategy in comparison to the PSA strategy. The primary cost saving was attributed to lower treatment expenses for metastatic disease. Sensitivity analysis affirmed the robustness of the findings across various diagnostic and treatment scenarios.

**Conclusions and clinical implications:**

Biomarker-enhanced diagnostic strategies may reduce health care costs for PC management and are likely to improve quality-adjusted life years in a scenario in which metastatic disease is reduced.

**Patient summary:**

We explored different ways to detect prostate cancer more cost-effectively. We found that using a specific blood test, called Stockholm3, after a PSA (prostate-specific antigen) test to decide if an MRI scan (magnetic resonance imaging) is necessary could save money, mainly by identifying localized cancer earlier and reducing the need for expensive treatments for advanced cancer.

## Take Home Message

Combining prostate-specific antigen (PSA) measurement with the Stockholm3 test in identifying candidates for magnetic resonance imaging in prostate cancer management could significantly reduce health care costs. This combined strategy outperformed the traditional PSA diagnostic approach. This cost reduction is primarily because of a decrease in costs related to metastatic prostate cancer.

## 1 Introduction

Prostate cancer (PC) is the most frequently diagnosed cancer among men in the European Union, accounting for 23.2% of all new cancer cases [1], and the third leading cause of cancer death, causing 9.9% of all male cancer fatalities [2]. PC is a costly health issue because of complex interventions, such as surgery, radiotherapy, and systemic therapy, as well as long-term follow-up and management of side effects and complications [3]. Consequently, there is an increasing focus on new diagnostic methods, including innovative biomarkers that, despite higher initial costs, could yield clinically advantageous outcomes [4] and reduce long-term health care costs.

Stockholm3 is a multiparametric test that incorporates five plasma protein markers, a polygenetic germline risk score, and clinical variables to predict the risk of clinically significant PC. It has been shown that use of Stockholm3 as an adjunct test to prostate-specific antigen (PSA) is effective in PC screening in large population-based trials and in real-world evidence, improving identification of curable clinically significant PC and reducing overdiagnosis of low-grade cancers [5], [6], [7], [8], [9]. The Stockholm3 test, while more costly upfront, offers potential economic benefits by enhancing the accuracy of PC detection and minimizing unnecessary diagnostic procedures.

The aim of this study was to explore cost implications in PC care associated with a biomarker-enhanced diagnostic strategy using Stockholm3. The study specifically focused on application of the Stockholm3 test as a reflex to PSA≥1.5 ng/ml in the context of European health care systems.

## 2 Material and methods

### 2.1 Diagnostic strategies

We assessed two diagnostic strategies for opportunistic testing (Supplementary Fig. 1):

(1) The PSA strategy starts with a PSA test, and PSA ≥3 ng/ml prompts a magnetic resonance imaging (MRI) scan. Prostate biopsy is recommended for a Prostate Imaging-Reporting and Data System (PI-RADS) scores ≥3.
(2) The biomarker-enhanced strategy starts with a PSA test, and a Stockholm3 test is performed in cases with PSA ≥1.5 ng/ml. A Stockholm3 score ≥11 prompts an MRI scan. Prostate biopsy is recommended for PI-RADS scores ≥3.

### 2.2 Model structure

Our model consists of a diagnostic decision tree and a health-state model. The diagnostic decision tree was used to calculate the costs of the diagnostic strategies. The health-state model was constructed as a Markov cohort model that describes the patterns of care for the disease groups over a 6-yr horizon and evaluates the effects of varying disease distributions presumed to result from the application of these diagnostic strategies over time.

Diagnostic probabilities were based on published data, and treatment probabilities were based on published data and expert opinion (Supplementary Table 1). The patient would either be categorized as healthy with no associated treatment costs, or fall into one of the four predefined disease states (Supplementary Fig. 2). The disease states were based on the 2014 International Society of Urological Pathology (ISUP) grade group classification and the tumor, node, metastasis (TNM) staging system as follows:

(1) Negative blood or imaging markers or benign biopsy (no cancer).
(2) Low-grade or clinically nonsignificant PC (ISUP 1).
(3) Intermediate- to high-grade localized or locally advance disease (ISUP 2–5).
(4) Metastatic disease (node-positive or metastatic disease).

The comparative efficacy of the PSA strategy and the biomarker-enhanced strategy was evaluated using real-world evidence and data from the Swedish National Prostate Cancer Register (NPCR; www.npcr.se/RATTEN), complemented by real-world cancer outcomes from Capio St. Göran Hospital (Supplementary Table 1), which integrated Stockholm3 into its diagnostic pathway [8]. Capio St. Göran Hospital tested 12 405 men using this pathway from 2018 to 2022 and represents the largest center to date using a Stockholm3-augmented diagnostic pathway. PSA referral for MRI was incorporated into the EAU guidelines [10] during the same period and was assumed to be part of standard of care. NPCR data from 2018–2022 showed that application of PSA ≥1.5 ng/ml, Stockholm3 ≥11, and MRI at the Capio Prostate Cancer Center resulted in stage migration in comparison to other hospitals in Sweden using PSA ≥3 ng/ml and MRI, with a 46% reduction in detection of ISUP 1 cancers, a 24% increase in identification of ISUP 2–5 cancers, and a 16% decrease in metastatic cancer detection [8]. It was assumed that diagnostic probabilities follow a steady-state system of interval PSA testing and capture a 6-yr cross-section.

### 2.3 Treatment strategies

In our model, all patients diagnosed with PC are assumed to receive care or treatment. The treatment modalities and health-stage transitions are summarized in Supplementary Table 2. Treatments were censored at 6 yr for patients in all disease groups.

For clinically nonsignificant ISUP 1 cancer, active surveillance was modeled for transition to curative treatment on disease progression, with 25% of patients reaching progression after 3 yr and 35% after 5 yr [11]. We assumed more diagnostic evaluations in the first year of active surveillance, with the frequency decreasing in subsequent years.

For clinically significant nonmetastatic ISUP 2–5 cancers, treatment included surgery or radiation therapy with androgen deprivation therapy (ADT), depending on the grade. To accommodate the difference between intermediate- and high-grade localized cancers, we estimated that treatments for patients with intermediate-grade disease would include radical prostatectomy (RP) or radiation therapy (RT) with an abbreviated course of ADT, whereas treatment for high-grade cancer would involve RP with pelvic lymphadenectomy, or RT with an extended course of ADT.

For men with metastatic PC (mPC), our approach was based on real-life treatment patterns [12], [13] reinforced by expert consensus. Treatment of metastatic disease included ADT, with systemic androgen receptor pathway inhibition (ARPI), chemotherapeutic agents, immune therapy, PARP inhibition, or radiopharmaceutical therapy. Osteoprotective medications were included as supportive care adjuncts to ADT for men with osteopenia, castration-resistant PC, or metastatic bone lesions, estimated to be 33% of all patients with metastatic disease. We assumed that 80% of patients treated for metastatic disease would receive ARPI therapies, with 25% of these also receiving triplet therapy including docetaxel, while the remaining 20% would receive taxane-based chemotherapy without an ARPI. Among all men with metastatic cancer, we assumed that 9% would receive a PARP inhibitor, 1% would receive immune therapy, and 30% would receive radiopharmaceutical therapy (50% ^177^Lu-labeled prostate-specific membrane antigen radioligand and 50% radium-223) in the final 2 yr.

We assumed a 30% rate for discontinuation of medication within the first year of diagnosis, increasing by 10% annually thereafter [14], [15]. Within 6 yr, approximately 50% of patients are expected to receive palliative care, and 35% are likely to receive end-of-life care [14], [15], [16], [17], [18].

### 2.4 Cost analysis

Both the treatment costs and patient outcomes based on a health sector perspective for selected European countries were attributed to the respective disease states in the model (Table 1). European costs for diagnostics and treatments were calculated using data from the French, German, Italian, Norwegian, Dutch, Swiss, UK, and Swedish health care systems. Cost data are reported in Euro and were gathered from the literature and expert opinion. The mean cost of treatment per patient was calculated for each of the disease states following the treatment model described in Supplementary Table 2. Future costs were discounted at 3% per year throughout the model to adjust to present-day values.

**Table 1:** Input costs for diagnostic and treatment options using average European health care costs consolidated from published data and expert opinion

Input	Value per unit/yr (SD)
Prostate-specific antigen test (including blood sampling)	€29 (€12)
Stockholm3 test	€400 (NA)
Magnetic resonance imaging (excluding radiology)	€406 (€169)
Biopsy (including pathology)	€1 347 (€1 540)
Radical prostatectomy	€10 832 (€3 464)
Radiation therapy	€7 726 (€3 334)
Pelvic lymphadenectomy	€1 500 (NA)^a^
ADT annual cost (average for degarelix, leurporelin, bicalutamide, nilutamide, flutamide)	€2 370 (€1 413)
ARPI annual cost (average for enzalutamide, apalutamide, darolutamide, abiraterone acetate)	€8 905 (€18 886)
Chemotherapy (docetaxel, cabazitaxel), full cycle cost	€15 519 (€9 476)
Immune therapy (pembrolizumab), annual cost	€104 264 (€28 046)
PARP inhibition (olaparaib/rucaparib), annual cost	€51 216 (€15 444)
Radiopharmaceutical therapy, annual cost	
^177^Lu-labeled prostate-specific membrane antigen radioligand	€90 106 (€36 598)
Radium-223	€25 692 (€5 825)
Prostate-specific membrane antigen positron emission tomography	€1 498 (€1 209)
Osteoprotective medication (zoledronic acid), annual cost	€1 058 (€1 031)
Palliative treatment (total cost)	€27 980 (€16 067)
End-of-life care (total cost)	€13 939 (€13 179)

ADT = androgen deprivation therapy; ARPI = androgen receptor pathway inhibitor; NA = not applicable; SD = standard deviation.

a The cost of pelvic lymphadenectomy was assumed to be €1500 on the basis of expert opinion, so SD is not available.

### 2.5 Model design and sensitivity analysis

A deterministic sensitivity analysis was performed to evaluate uncertainties in costs and diagnostic probabilities. This analysis examined minimum and maximum diagnostic costs. For the sensitivity analysis, the upper and lower estimates of treatment costs and disease-state probabilities were evaluated at values 30% higher and lower than the baseline values used.

### 2.6 Comparison with PC risk calculators

A subanalysis using a clinical risk calculator, the Rotterdam Prostate Cancer Risk Calculator (SWOP), in the PSA strategy for MRI referral was performed. Diagnostic and treatment probabilities were based on published data [19] (Supplementary Table 1). The mPC group mirrored the PSA + MRI strategy, with an additional proportion (10%) of the SWOP false-negative cases projected to progress to metastatic disease within 6 yr on the basis of long-term data from the PIVOT trial [20]. The added cost of SWOP risk estimation included the cost of transrectal ultrasound measurement of prostate volume (€100).

### 2.7 Statistical analysis

The Mann-Whitney *U* test was used to assess differences in cost outcomes between the suggested pathways for PC detection cancer at a two-tailed significance level of α = 0.05.

## 3 Results

Including the costs of diagnostic procedures, the mean expenditure associated with the four disease states was as follows: €2 182 for benign disease, €10 023 for low-grade disease, €13 073 for intermediate- to high-grade localized disease, and €271 210 for metastatic disease.

**Fig. 1:**
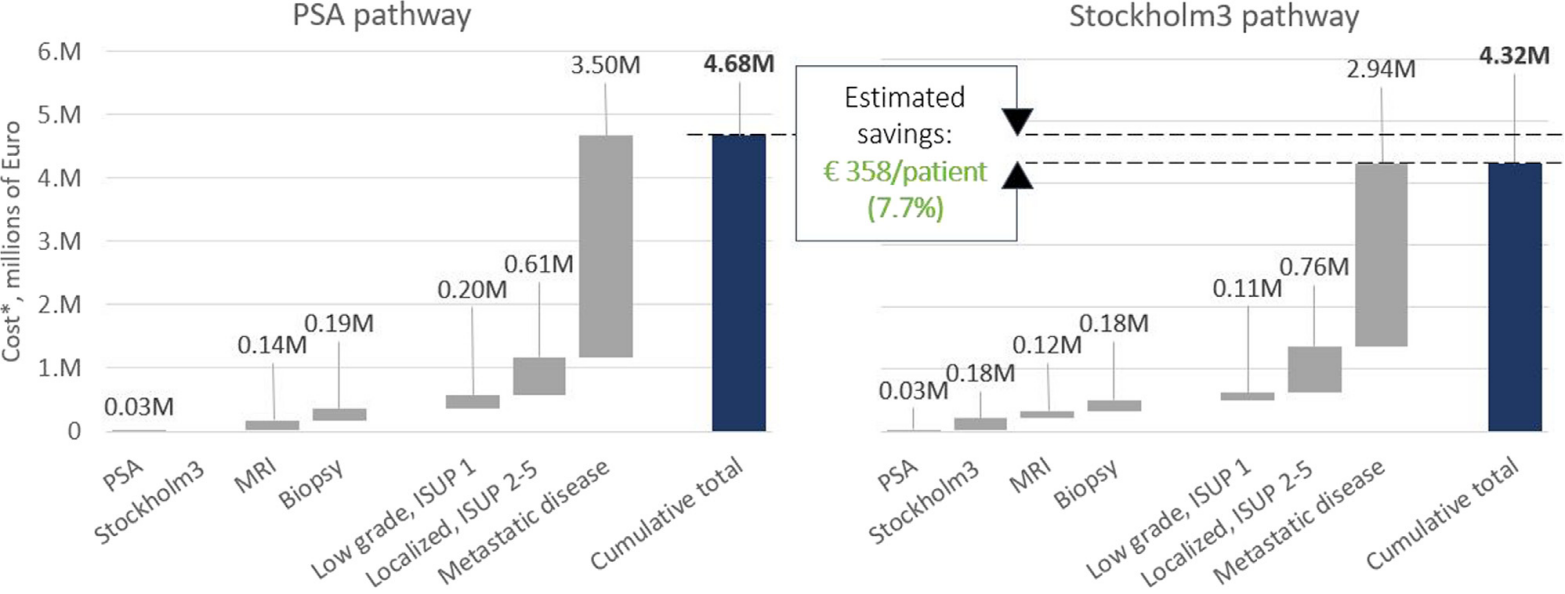
Cost effects of Stockholm3 implementation in the prostate cancer care pathway. ISUP = International Society of Urological Pathology grade group; MRI = magnetic resonance imaging; PSA = prostate-specific antigen.

The diagnostic accuracy results for the different strategies are reported in Supplementary Table 1. The PSA strategy resulted in an estimated overall cost of €4.68 million per 1000 men tested (average €4 676 per man), whereas the enhanced biomarker strategy resulted in an estimated overall cost of €4.32 million per 1000 men tested (average €4 318 per man). The diagnostic costs were €151 (42%) higher on average per man tested with the biomarker-enhanced strategy. Stockholm3 contributed 35% to the total cost of diagnostics. There was an overall net saving of €358 239 (7.7%), or €358 per man tested, with the biomarker-enhanced strategy in comparison to the PSA strategy (*p* < 0.001; Fig. 1). The largest driver of cost savings in the model was treatment of metastatic disease, accounting for €3.50 million in PSA arm versus €2.94 million in the biomarker-enhanced arm.

**Fig. 2:**
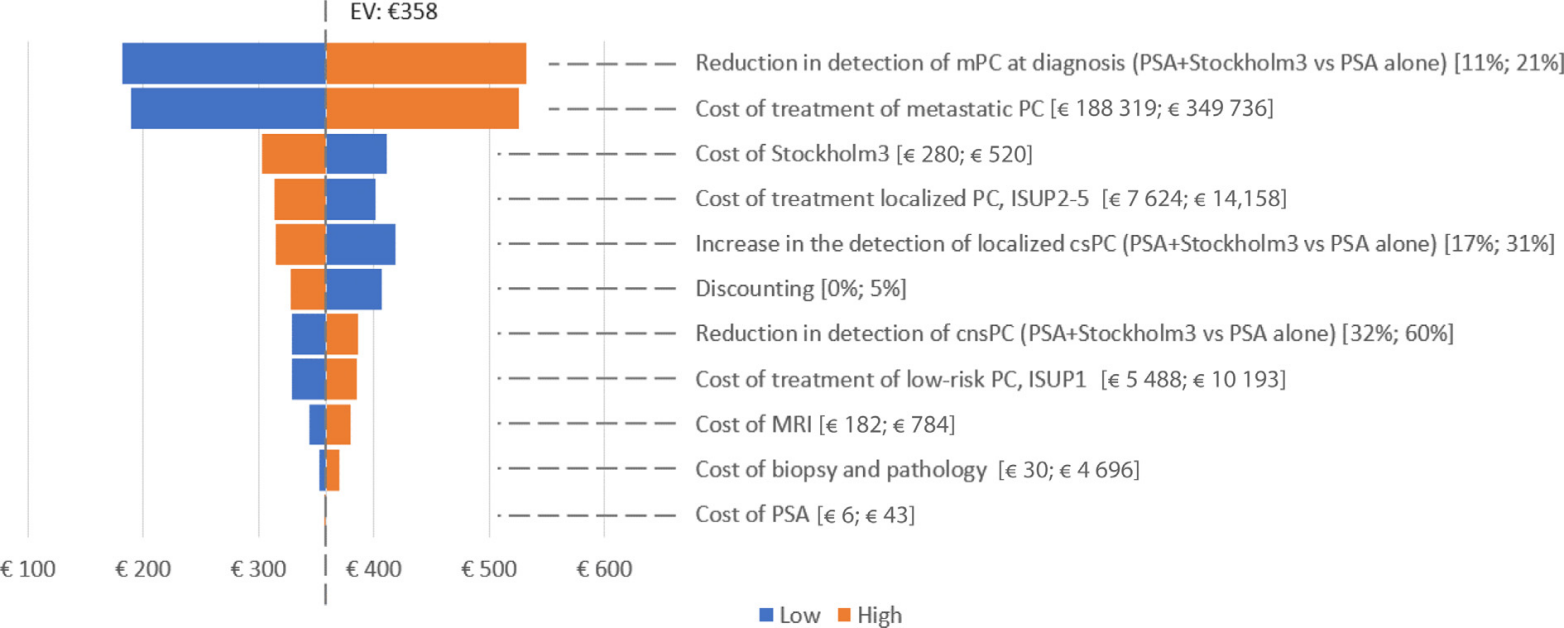
Tornado diagram for the one-way deterministic sensitivity analysis: comparison between the PSA arm and the Stockholm3 arm. csPC = clinically significant PC; cnsPC = clinically nonsignificant PC; EV = expected value; ISUP = International Society of Urological Pathology grade group; MRI = magnetic resonance imaging; mPC = metastatic PC; PC = prostate cancer; PSA = prostate-specific antigen.

One-way sensitivity analysis showed that the cost-saving effect of the biomarker-enhanced strategy persisted across various scenarios (Fig. 2). The most influential parameter was the cost of treatment for metastatic disease, followed by the proportion of men presenting with mPC at diagnosis. Our analysis estimated savings ranging from €189 to €525 per individual tested when using treatment costs that were 30% lower (€188 319) and 30% higher (€349 736). A 6.5-yr evaluation showed that the Stockholm3 strategy would reduce detection of advanced disease by 16%, with sensitivity analysis indicated cost savings ranging from €182 to €532 per person screened for variation of the net decrease in mPC at presentation from 11% to 21%.

The total treatment costs associated with the SWOP-adapted pathway amount to €4 787 048 per 1000 men, which is 2.4% higher than the PSA pathway (*p* = 0.006) and 10.9% higher than the Stockholm3-enhanced pathway (*p* < 0.001). Although SWOP would achieve diagnostic cost reductions of 43% and 60% in comparison to the PSA and Stockholm3-enhanced pathways, mPC treatment costs would increase by 6% and 20%, respectively, over these pathways (Supplementary Table 3).

## 4 Discussion

Previous studies have shown the clinical utility of a low PSA threshold of 1.5 ng/ml combined with the Stockholm3 blood biomarker test in enhancing the detection of curable PC [7], [8], [9]. Our analysis indicates that this approach would also offer a cost saving of €358 per individual assessed in comparison to PSA-and MRI-based detection methods, which would translate into marked annual savings for the populations in European countries. These savings are predominantly because of reduced expenditure associated with stage migration of mPC to localized PC observed from NPCR data when Stockholm3 is used as an adjunct test to PSA≥1.5 ng/ml.

Other well-validated blood and urinary biomarkers used after elevated PSA have the advantage of a reduction in downstream diagnostic tests, including MRI and biopsy. Several of these biomarkers have been evaluated for cost effectiveness, including the Prostate Health Index [21], 4Kscore [22], PSE EpiSwitch [23], and SelectMDx [24]. Studies have shown that these biomarkers are cost effective because they reduce unnecessary follow-up while retaining good relative sensitivity comparable to PSA, supporting their use for more efficient PC cancer detection. Similarly, risk calculators can be used for risk stratification following PSA testing. The SWOP risk calculator is a well-documented example and can decrease the number of MRI scans and biopsies performed [19], [25], [26], [27]. These strategies improve resource utilization, but this may come at the expense of missing clinically aggressive cancers that lead to more downstream costs, as seen in this analysis with the SWOP risk calculator. Despite good evidence regarding a reduction in unnecessary testing in comparison to PSA ≥3 ng/ml, the challenge is detection of aggressive, low–PSA-producing cancers. By contrast, Stockholm3 provides evidence for detection of such cancers and represents a robust alternative for early detection [8], [9], [28].

Our Markov model is predicated on key assumptions, notably the diagnostic efficacy of PSA versus biomarker-enhanced strategies, which we obtained from real-world data. Real-world data from Capio St. Göran Hospital (2018–2022) demonstrate that Stockholm3 reduces MRI use by 53% for men with PSA ≥3 ng/ml [8]. In the same center, more PC cases were detected among men with PSA in the range 1.5–3 ng/ml, which allows earlier detection of curable cases of clinically significant PC, and probably reduces diagnoses of advanced PC, as supported by long-term data. The feasibility of observing impacts on mPC detection over a period of 6 yr may be debatable because of potential changes in diagnostic and treatment pathways.

Our study has several strengths, including the real-world registry data used to define the distribution of disease states in the different diagnostic strategies. Despite the absence of quality-adjusted life year (QALY) measurements in our study, the reduction in metastatic disease observed in real-world evidence studies [5], [6], [7] implies an additional improvement in QALYs. Consequently, it is reasonable to infer that the model would demonstrate further benefits when evaluating QALYs concurrent with reduced costs, thereby establishing a dominant cost-benefit scenario. Karlsson et al [29] and Hao et al [30] assessed the cost effectiveness of PSA and Stockholm3 testing in comparison to PSA alone in both biopsy-alone and MRI + biopsy settings. These studies used lifetime societal perspective costs and considered both direct and indirect costs in the setting of diagnostic screening by invitation; however, they did not include treatment-related costs for localized or metastatic cancers. By contrast, our analysis includes real-world opportunistic testing (NPCR data) and treatment-related costs. Furthermore, we incorporated European cost structures, whereas previous studies evaluated only Swedish cost structures.

Despite its strengths, our analysis has several limitations. Assumptions for disease states were based on a 6-yr horizon in Sweden, which may not be generalizable to other nations with different PC profiles. The model censored data at 6 yr, which meant further downstream costs, specifically in the metastatic group, were not included. Medicine costs are subject to change because of factors such as patent expiration, and this may affect the cost analysis in future studies. Although age is included in the Stockholm3 algorithm, the models do not account for age, and do not include adverse effects, comorbidities, or indirect or intangible costs, such as lost productivity. Although the model considers discontinuation yearly, it assumes complete compliance at treatment initiation, and does not take potential noncompliance into account. Our study relies on accuracies determined from Swedish PC registries, and intergroup differences related to racial and ethnic disparities in the costs of PC care are not considered in this evaluation, as in other studies [31]. However, Stockholm3 has been evaluated in different ethnic groups [32], with similar performance for Asian, Black, and Hispanic men in comparison to White men, and in this study we assumed equal care across the study population modeled.

## 5 Conclusions

Implementation of a biomarker-enhanced strategy that integrates PSA testing with the Stockholm3 test for selection of patients for MRI could reduce health care costs in prostate cancer management. This approach markedly outperformed the PSA diagnostic strategy with a threshold of ≥3 ng/ml to determine the need for MRI. These findings were largely driven by the reduction in costs for metastatic prostate cancer.

  ***Author contributions:*** Olga Dianna McLeod had full access to all the data in the study and takes responsibility for the integrity of the data and the accuracy of the data analysis.

  *Study concept and design*: McLeod, Palsdottir, Walz, Tilki, Briganti, Stabile, Nyre Vigmostad, Mortezavi, Elyan, Dudderidge, Govers, Grönberg, Vigneswaran.

*Acquisition of data*: McLeod, Palsdottir, Walz, Tilki, Briganti, Stabile, Nyre Vigmostad, Mortezavi, Elyan, Dudderidge, Govers, Grönberg, Vigneswaran.

*Analysis and interpretation of data*: McLeod, Palsdottir, Walz, Tilki, Briganti, Stabile, Nyre Vigmostad, Mortezavi, Elyan, Dudderidge, Govers, Grönberg, Vigneswaran.

*Drafting of the manuscript*: McLeod, Palsdottir, Vigneswaran.

*Critical revision of the manuscript for important intellectual content*: McLeod, Palsdottir, Walz, Tilki, Briganti, Stabile, Nyre Vigmostad, Mortezavi, Elyan, Dudderidge, Govers, Grönberg, Vigneswaran.

*Statistical analysis*: McLeod, Palsdottir.

*Obtaining funding*: Grönberg.

*Administrative, technical, or material support*: None.

*Supervision*: None.

*Other*: None.

  ***Financial disclosures:*** Olga Dianna McLeod certifies that all conflicts of interest, including specific financial interests and relationships and affiliations relevant to the subject matter or materials discussed in the manuscript (eg, employment/affiliation, grants or funding, consultancies, honoraria, stock ownership or options, expert testimony, royalties, or patents filed, received, or pending), are the following: Olga Dianna McLeod, Thorgerdur Palsdottir, and Hari Vigneswaran reported employment at A3P Biomedical. Ashkan Mortezavi, Derya Tilki, and Alberto Briganti have received consulting fees from A3P Biomedical. Jochen Walz has received consulting fees from A3P, ANNA/C-TRUS, and 3D Biopsy, and research funding from Exact Imaging. Tim Govers reports stock ownership in Medip Analytics, a company that provides services to A3P Biomedical. Henrik Grönberg reports consulting fees from Bayer, Astellas, and AstraZeneca, stock ownership and patent interests in A3P Biomedical, research funding from Janssen, and five pending patents related to prostate cancer diagnostics (WO0213EP74259201311120, WO2013EP7427020131120, WO2018EP5247320180201, WO2013SE5055420130516, AND WO2015SE5027220150311). The remaining authors have nothing to disclose.

  ***Funding/Support and role of the sponsor:*** This work was supported by the Swedish Research Council and the Swedish Cancer Society. The sponsors played no direct role in the study.
