# Supplementary material for: Cost Analysis of Prostate Cancer Care Using a Biomarker-enhanced Diagnostic Strategy with Stockholm3
Source: Eur Urol Open Sci. 2024 Jun 25;66:26–32. doi: 10.1016/j.euros.2024.05.010 (PMC11254591; doi:10.1016/j.euros.2024.05.010)

**Supplementary Figure 1. Diagnostic decision tree model for the compared study strategies – PSA vs Stockholm3 strategy (main design).**


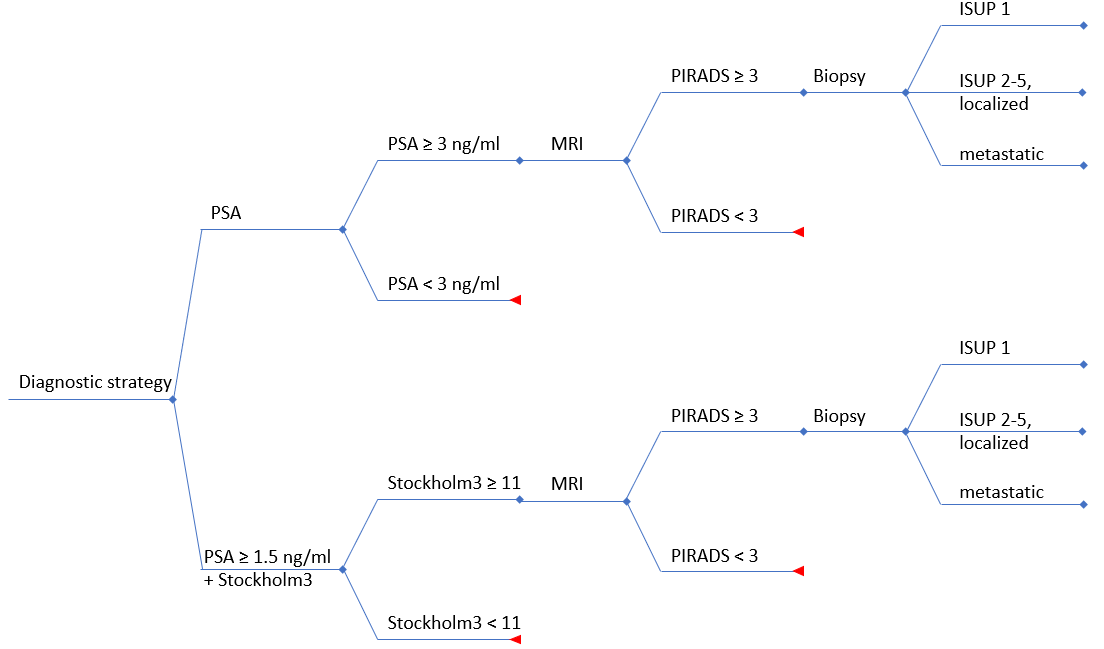


**Supplementary Figure 2.** **Simplified schematic of the key disease states in the health state model.**


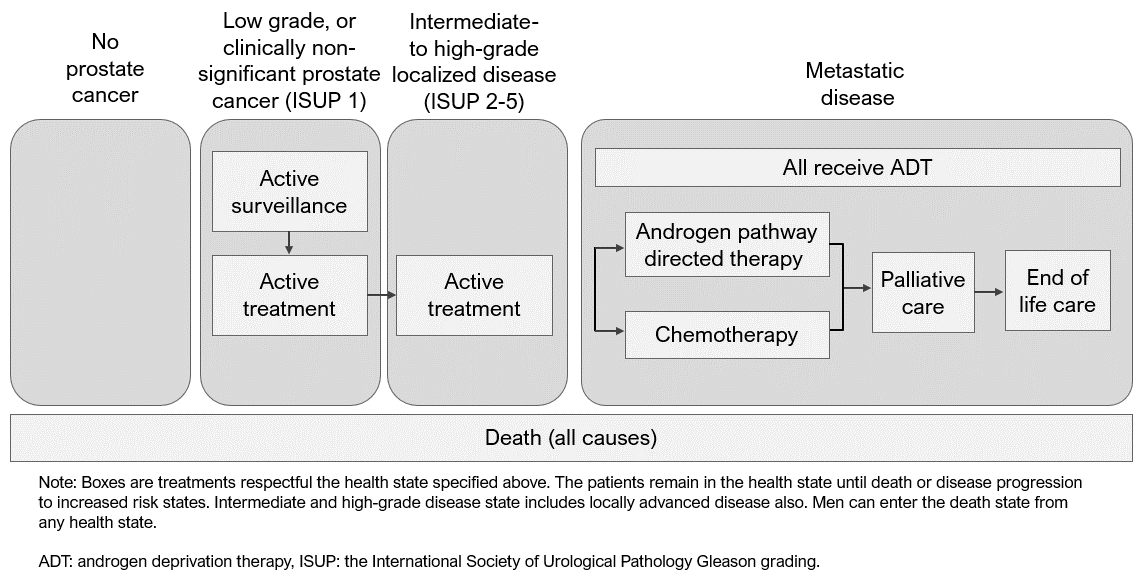


**Supplementary Figure 3. Diagnostic decision tree model for the complementary comparison with the Rotterdam risk calculator (SWOP) – PSA to SWOP to MRI vs PSA to Stockholm3 to MRI.**


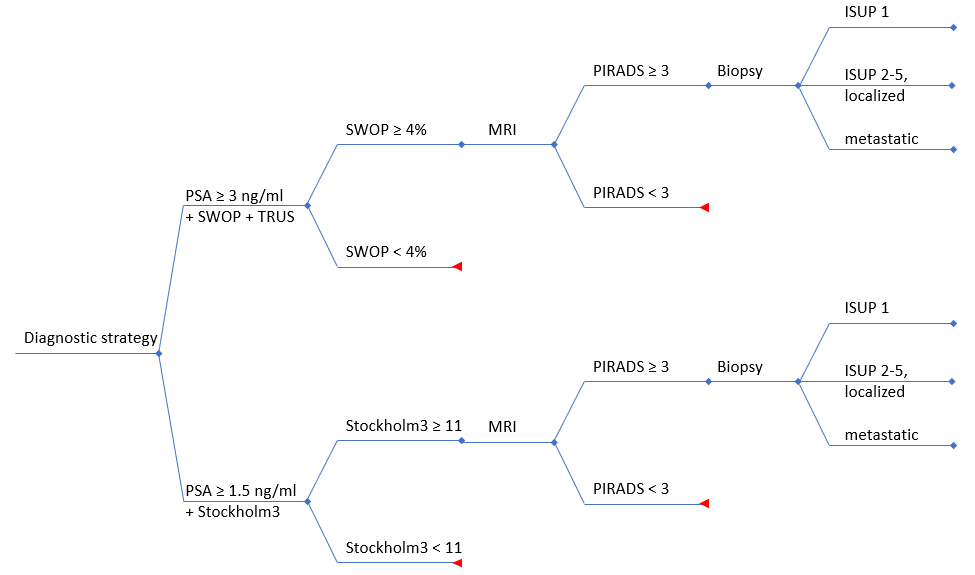

Supplement: Supplementary figures [file mmc1.docx]
