# Supplementary material for: Cost Analysis of Prostate Cancer Care Using a Biomarker-enhanced Diagnostic Strategy with Stockholm3
Source: Eur Urol Open Sci. 2024 Jun 25;66:26–32. doi: 10.1016/j.euros.2024.05.010 (PMC11254591; doi:10.1016/j.euros.2024.05.010)
Supplement: Supplementary tables [file mmc2.docx]

**Supplementary Table 1. Key model inputs and structural assumptions.**

| **Input** | **Value(s)** | **Source / Rationale** |
| --- | --- | --- |
| Cohort age | Men aged 45-74 years with a mean of 67 years. A normal distribution applied. | Median age of men diagnosed with prostate cancer is often reported as 67 years. |
| Time horizon | 6 years | A 6-year time horizon enhances feasibility and reduces uncertainty by minimizing the need for data extrapolation. This is particularly relevant in a rapidly evolving therapeutic landscape where new treatments could potentially improve outcomes and alter cost structures. |
| Discounting | 3% costs, annually | In Europe, accepted discounting rates vary between 2.5% and 5%, with 3% being the most common ^1^ |
| Costs | Mean between included countries | The transparency of drug costs is often obscured both within and between the countries, exacerbated by other factors, such as rebates and discounts. To address this, we used the averaged costs across the dataset. |
| **Diagnostic probabilities** | | |
| PSA ≥ 3 ng/ml | 35% | Published data ^2^ |
| PSA ≥ 1.5 ng/ml | 50% | Published data ^2^ |
| Elevated risk defined by Stockholm3 ≥ 11 | 29% | Published data ^2^ |
| Elevated risk defined by SWOP ≥ 4% | 13% | Published data ^3^ |
| Share positive MRI (PIRADS ≥ 3) using PSA ≥ 3 ng/ml | 40% | Published data ^2,3^ |
| Share positive MRI (PIRADS ≥ 3) using Stockholm3 ≥ 11 and/or SWOP ≥ 4% | 47% | Published data ^2,3^ |
| **Results of prostate biopsy:** | | |
| *PSA strategy:* | | |
| No malignancy | 30% | Published data^4-9^ and expert opinion |
| cnsPC, low grade (ISUP 1) | 20% | Published data^4-9^ and expert opinion |
| Localized csPC (ISUP 2-5) | 40% | Published data^5-9^ and expert opinion |
| Advanced PC (node positive or metastatic disease) | 10% | Published data ^5-9^ and expert opinion |
| *Biomarker-enhanced strategy:* | | |
| Benign biopsy – biomarker-enhanced  (Δ probability of +4% *) | 34% | Published data ^2^ |
| cnsPC, low grade (ISUP 1) – biomarker-enhanced  (Δ probability of -46% *) | 11% | Published data ^2^ |
| Localized csPC (ISUP 2-5) – biomarker-enhanced  (Δ probability of +24% *) | 50% | Published data ^2^ |
| Advanced or metastatic PC – biomarker-enhanced  (Δ probability of -16% *) | 8% | Published data ^2^ |
| *SWOP-enhanced strategy:* | | |
| Benign biopsy – PSA + SWOP-enhanced | 35% | Published data ^3^ |
| cnsPC, low grade (ISUP 1) - SWOP-enhanced | 12% | Published data ^3^ |
| Localized csPC  (ISUP 2-5) –  SWOP-enhanced | 53% | Published data ^3^ |
| Advanced or metastatic PC - SWOP-enhanced | 24% | Estimate based on published data ^3,10^ |
| *Missed cancers in SWOP-enhanced strategy:* | | |
| Probability of developing metastases in untreated intermediate- to high-risk PC within 6 years | 10% | Published data ^10^ |
| **Treatment probabilities** | | |
| AS in low grade disease | 100% | Expert opinion |
| Men transitioning from AS to active treatment  (4 years after diagnosis, total) | 25% | Expert opinion |
| Men transitioning from AS to active treatment  (6 years after diagnosis, total) | 35% | Expert opinion |
| Men with localized csPC (ISUP 2-5) undergoing treatment | 100% | Expert opinion |
| RP in intermediate grade localized disease | 50% | Expert opinion |
| PLD in intermediate grade localized disease (of those treated with RP) | 25% | Expert opinion |
| RT in intermediate grade localized disease | 50% | Expert opinion |
| ADT in intermediate grade localized disease (of those treated with RT) | 25% | Expert opinion |
| RP + PLD in high grade localized disease | 50% | Expert opinion |
| RT + ADT in high grade localized disease | 50% | Expert opinion |
| ADT in advanced or metastatic PC | 100% | Expert opinion |
| ARPI in advanced or metastatic PC | 80% | Expert opinion |
| Triplet therapy in metastatic PC | 20% | Expert opinion |
| PARP inhibition in metastatic PC | 9% | Expert opinion |
| Immune therapy in metastatic PC | 1% | Expert opinion |
| 177Lu-PSMA in metastatic PC | 15% | Expert opinion |
| Radium-223 in metastatic PC | 15% | Expert opinion |
| Chemotherapy (taxanes) in advanced or metastatic PC | 20% | Expert opinion |
| Men with advanced or metastatic PC receiving osteoprotective medication | 33% | Expert opinion |
| Palliative treatment in advanced or metastatic PC after 6 years | 50% | Expert opinion |
| Patients with advanced or metastatic PC requiring end-of-life care after 6 years | 35% | Literature review^11-14^ and expert opinion |
| **Probability of recurrence and progression** | | |
| Progression to localized disease in low grade  (3 years after diagnosis) | 25% | Literature review ^15^ and expert opinion |
| Progression to localized disease in low grade (within 6 years after diagnosis) | 35% | Literature review ^16^ and expert opinion |
| Bone metastases in advanced disease | 30-60% | Literature review ^17,18^ and expert opinion |
| Overall survival in advanced or metastatic disease within 6 years | 50% | Literature review ^13,19-22^ and expert opinion |

*Change of diagnostic probability estimated as an effect of PSA-Stockholm3-MRI diagnostic pathway as compared to PSA-MRI pathway.

ADT: androgen deprivation therapy, ARPI: androgen receptor pathway inhibitor, AS: active surveillance, cnsPC: clinically non-significant prostate cancer, csPC: clinically significant prostate cancer, MRI: magnetic resonance imaging, PC: prostate cancer, PLD: pelvic lymphadenopathy, PSA: prostate specific antigen, RP: radical prostatectomy, RT: radiation therapy

**Supplementary Table 2: Breakdown of treatment cost components* across the treatment groups following the prostate cancer diagnosis.**

| Clinically non-significant “low grade” PC (ISUP 1) | Clinically significant, localized PC (ISUP 2-5) | Advanced or metastatic PC |
| --- | --- | --- |
| Year 1: PSA x2 + MRI x0.5 (100%)  Year 2: PSA x2 + MRI x0.5 + Biopsy x1 (100%)  Year 3: PSA x2 + MRI x0.5 (100%)  Year 4: PSA x2 + MRI x0.5 + Biopsy x1 (75%),  RP or RT (25%)  Year 5: PSA x2 + MRI x0.5 + Biopsy x1 (75%)  Year 6: PSA x2 + MRI x0.5 (65%)  RP or RT (10%) | Intermediate grade (ISUP 2-3):  50%: RP (+ 25% of RPs are performed with PLD)  50%: RT (+ 25% of patients undergoing RT will be administered ADT for 6 months);  Monitoring: PSA x12 | ADT – continuous 6 years (100%)  ARPI – continuous 6 years (40%) with 50% of these patients receiving triplet therapy  ARPI switch after 4 years (40%) to:  PARP inhibitors – last 2 years (9%)  Immune therapy – last 2 years (1%)  177Lu-PSMA – last 2 years (15%)  Radium-223 – last 2 years (15%)  Osteoprotection medication – continuous 6 years (33%)  ChT (docetaxel, cabazitaxel) – full cycle treatment (20%)  Monitoring (100%):  PSMA PET-CT scan - x2 under 6 years  PSA x12 under 6 years  Palliative care (50%) including RT x1  End of life care (35%) |
|  | High grade (ISUP 4-5):  50%: RP + PLD  50%: RT with 24 months ADT;  Monitoring: PSA x12 |  |
| **€ 7,840** | **€ 10,891** | **€ 269,397** |

* Individual procedure cost estimates are presented in Table 1.

Assumptions: Men aged 45-75 years. Costs for PSA and Stockholm3 analysis, MRI, biopsy and pathology workup and treatments are taken into account, but complications are excluded. Costs are estimated using prices from French, German, Italian, Norwegian, Dutch, Swiss, UK and Swedish healthcare systems. Sensitivity and specificity of PSA and Stockholm3 and outcome in biopsy were derived from Palsdottir et al. (2024). The estimated total cost for localized PC is based on an average between the treatment costs for intermediate and high-grade groups, considering the intermediate risk group is twice as large as the high-risk group. Time horizon was censored to 6 years.

ADT: androgen deprivation therapy, ARPI: androgen receptor pathway inhibitor, ChT: chemotherapy, MRI: magnetic resonance imaging, PARP inhibitor: Poly (ADP-ribose) polymerase inhibitor therapy, PLD: pelvic lymphadenectomy, PSA: prostate specific antigen, PSMA PET-CT: prostate-specific membrane antigen positron emission tomography scan, RP: radical prostatectomy, RT: radiotherapy.

**Supplementary Table 3. Cost comparison of PSA, Stockholm3, and SWOP pathways in prostate cancer diagnostics and treatment (per 1,000 men tested).**

| **Diagnostic CostsColumn1** | | | | **Column2** | | | **Column3** | | | | | **Column4** | | **Column5** | | | | | | | **Column6** | | | | | |  |  |  |
| --- | --- | --- | --- | --- | --- | --- | --- | --- | --- | --- | --- | --- | --- | --- | --- | --- | --- | --- | --- | --- | --- | --- | --- | --- | --- | --- | --- | --- | --- |
| **Pathway** | **PSA** | | **Stockholm3** | | | **SWOP** | | | | | **MRI** | | | | **Biopsy** | | | | | **Total diagnostic cost** | | | | **Cost Difference (PSA)** | | **Cost Difference (Stockholm3)** | | |  |
| PSA | € 29,018 | | - | | | - | | | | | € 142,160 | | | | € 188,620 | | | | | € 359,798 | | | | - | |  | | |  |
| Stockholm3 | € 29,018 | | € 180,000 | | | - | | | | | € 117,790 | | | | € 183,635 | | | | | € 510,443 | | | | **€ 150,645 (42%)** | |  | | |  |
| SWOP | € 29,018 | | - | | | € 35,000 | | | | | € 52,599 | | | | € 87,039 | | | | | € 203,656 | | | | **€ -156,141 (-43%)** | | **-€ 306,786 (-60%)** | | |  |
|  |  | | |  | | | |  | | | | |  | | | | |  | | | | | |  | | | | |  |
| **Treatment costs** | | | |  | | | |  | | | | |  | | | | |  | | | | | |  | | | | |  |
| **Pathway** | **cnsPC** | | | | **csPC** | | | | | **mPC** | | | | | | | **Total  treatment cost** | | | | | **Cost Difference (PSA)** | | | **Cost Difference (Stockholm3)** | | |  |  |
| PSA | € 204,153 | | | | € 609,897 | | | | | € 3,507,557 | | | | | | | € 4,316,608 | | | | | - | | | - | | |  |  |
| Stockholm3 | € 109,304 | | | | € 756,272 | | | | | € 2,942,148 | | | | | | | € 3,807,724 | | | | | **-€ 508,884 (-12%)** | | | - | | |  |  |
| SWOP | € 24,498 | | | | € 320,379 | | | | | € 4,238,515 | | | | | | | € 4,583,392 | | | | | **€ 266,784 (6.2%)** | | | **€ 775,668 (20%)** | | |  |  |
|  |  | | |  | | | |  | | | | |  | | | | |  | | | | | |  | | |  |  |  |
| **Total Costs** | |  | |  | | |  | | | | |  | |  | | | | | | |  | | | | | |  |  |  |
| **Pathway** | | **Total** | | **Cost Difference (PSA)** | | | | | **Cost Difference (Stockholm3)** | | | | | | |  | | |  | | | |  | | | | | | |
| PSA | | 4,676,405 | | - | | | | | - | | | | | | |  | | |  | | | |  | | | | | | |
| Stockholm3 | | 4,318,167 | | **-€ 358,239 (-7.7%)** | | | | | - | | | | | | |  | | |  | | | |  | | | | | | |
| SWOP | | 4,787,048 | | **€ 110,643 (2.4%)** | | | | | **€ 468,882 (10.9%)** | | | | | | |  | | |  | | | |  | | | | | | |

1. Khorasani E, Davari M, Kebriaeezadeh A, Fatemi F, Akbari Sari A, Varahrami V. A comprehensive review of official discount rates in guidelines of health economic evaluations over time: the trends and roots. *Eur J Health Econ*. Dec 2022;23(9):1577-1590. doi:10.1007/s10198-022-01445-x

2. Palsdottir T, Soderback H, Jaderling F, Bergman M, Vigneswaran H, Gronberg H. The Capio Prostate Cancer Center Model for Prostate Cancer Diagnostics-Real-world Evidence from 2018 to 2022. *Eur Urol Open Sci*. Mar 2024;61:29-36. doi:10.1016/j.euros.2024.01.012

3. Palsdottir T, Gronberg H, Hilmisson A, Eklund M, Nordstrom T, Vigneswaran HT. External Validation of the Rotterdam Prostate Cancer Risk Calculator and Comparison with Stockholm3 for Prostate Cancer Diagnosis in a Swedish Population-based Screening Cohort. *Eur Urol Focus*. May 2023;9(3):455-462. doi:10.1016/j.euf.2022.11.021

4. Camacho A, Salah F, Bay CP, et al. PI-RADS 3 score: A retrospective experience of clinically significant prostate cancer detection. *BJUI Compass*. Jul 2023;4(4):473-481. doi:10.1002/bco2.231

5. Evans SM, Millar JL, Wood JM, et al. The Prostate Cancer Registry: monitoring patterns and quality of care for men diagnosed with prostate cancer. *BJU Int*. Apr 2013;111(4 Pt B):E158-66. doi:10.1111/j.1464-410X.2012.11530.x

6. Kaufmann B, Saba K, Schmidli TS, et al. Prostate cancer detection rate in men undergoing transperineal template-guided saturation and targeted prostate biopsy. *Prostate*. Feb 2022;82(3):388-396. doi:10.1002/pros.24286

7. Panebianco V, Barchetti F, Sciarra A, et al. Multiparametric magnetic resonance imaging vs. standard care in men being evaluated for prostate cancer: a randomized study. *Urol Oncol*. Jan 2015;33(1):17 e1-17 e7. doi:10.1016/j.urolonc.2014.09.013

8. Mannaerts CK, Gayet M, Verbeek JF, et al. Prostate Cancer Risk Assessment in Biopsy-naive Patients: The Rotterdam Prostate Cancer Risk Calculator in Multiparametric Magnetic Resonance Imaging-Transrectal Ultrasound (TRUS) Fusion Biopsy and Systematic TRUS Biopsy. *Eur Urol Oncol*. Jun 2018;1(2):109-117. doi:10.1016/j.euo.2018.02.010

9. Nordstrom T, Discacciati A, Bergman M, et al. Prostate cancer screening using a combination of risk-prediction, MRI, and targeted prostate biopsies (STHLM3-MRI): a prospective, population-based, randomised, open-label, non-inferiority trial. *Lancet Oncol*. Sep 2021;22(9):1240-1249. doi:10.1016/S1470-2045(21)00348-X

10. Wilt TJ, Jones KM, Barry MJ, et al. Follow-up of Prostatectomy versus Observation for Early Prostate Cancer. *N Engl J Med*. Jul 13 2017;377(2):132-142. doi:10.1056/NEJMoa1615869

11. Davis ID, Martin AJ, Zielinski RR, et al. Updated overall survival outcomes in ENZAMET (ANZUP 1304), an international, cooperative group trial of enzalutamide in metastatic hormone-sensitive prostate cancer (mHSPC). *Journal of Clinical Oncology*. 2022;40(17_suppl):LBA5004-LBA5004. doi:10.1200/JCO.2022.40.17_suppl.LBA5004

12. Shen J, Chowdhury S, Agarwal N, et al. Apalutamide efficacy, safety and wellbeing in older patients with advanced prostate cancer from Phase 3 randomised clinical studies TITAN and SPARTAN. *Br J Cancer*. Jan 2024;130(1):73-81. doi:10.1038/s41416-023-02492-8

13. Armstrong AJ, Azad AA, Iguchi T, et al. Improved Survival With Enzalutamide in Patients With Metastatic Hormone-Sensitive Prostate Cancer. *J Clin Oncol*. May 20 2022;40(15):1616-1622. doi:10.1200/JCO.22.00193

14. Attard G, Murphy L, Clarke NW, et al. Abiraterone acetate and prednisolone with or without enzalutamide for high-risk non-metastatic prostate cancer: a meta-analysis of primary results from two randomised controlled phase 3 trials of the STAMPEDE platform protocol. *Lancet*. Jan 29 2022;399(10323):447-460. doi:10.1016/S0140-6736(21)02437-5

15. San Francisco IF, Werner L, Regan MM, Garnick MB, Bubley G, DeWolf WC. Risk stratification and validation of prostate specific antigen density as independent predictor of progression in men with low risk prostate cancer during active surveillance. *J Urol*. Feb 2011;185(2):471-6. doi:10.1016/j.juro.2010.09.115

16. Hamdy FC, Donovan JL, Lane JA, et al. Fifteen-Year Outcomes after Monitoring, Surgery, or Radiotherapy for Prostate Cancer. *N Engl J Med*. Apr 27 2023;388(17):1547-1558. doi:10.1056/NEJMoa2214122

17. Smith MR, Kabbinavar F, Saad F, et al. Natural history of rising serum prostate-specific antigen in men with castrate nonmetastatic prostate cancer. *J Clin Oncol*. May 1 2005;23(13):2918-25. doi:10.1200/JCO.2005.01.529

18. Qian Y, Arellano J, Gatta F, et al. Physicians' preferences for bone metastases treatments in France, Germany and the United Kingdom. *BMC Health Serv Res*. Jul 3 2018;18(1):518. doi:10.1186/s12913-018-3272-x

19. Fizazi K, Tran N, Fein L, et al. Abiraterone acetate plus prednisone in patients with newly diagnosed high-risk metastatic castration-sensitive prostate cancer (LATITUDE): final overall survival analysis of a randomised, double-blind, phase 3 trial. *Lancet Oncol*. May 2019;20(5):686-700. doi:10.1016/S1470-2045(19)30082-8

20. Chi KN, Agarwal N, Bjartell A, et al. Apalutamide for Metastatic, Castration-Sensitive Prostate Cancer. *N Engl J Med*. Jul 4 2019;381(1):13-24. doi:10.1056/NEJMoa1903307

21. Damodaran S, Kyriakopoulos CE, Jarrard DF. Newly Diagnosed Metastatic Prostate Cancer: Has the Paradigm Changed? *Urol Clin North Am*. Nov 2017;44(4):611-621. doi:10.1016/j.ucl.2017.07.008

22. Orrason AW, Garmo H, Styrke J, Dickman PW, Stattin P. Comparison of Relative Survival and Cause-Specific Survival in Men With Prostate Cancer According to Age and Risk Category: A Nationwide, Population-Based Study. *Am J Epidemiol*. Oct 1 2021;190(10):2053-2063. doi:10.1093/aje/kwab146
